# Visualising peripheral arterioles and venules through high-resolution and large-area photoacoustic imaging

**DOI:** 10.1038/s41598-018-33255-8

**Published:** 2018-10-08

**Authors:** Yoshiaki Matsumoto, Yasufumi Asao, Hiroyuki Sekiguchi, Aya Yoshikawa, Tomoko Ishii, Ken-ichi Nagae, Shuichi Kobayashi, Itaru Tsuge, Susumu Saito, Masahiro Takada, Yoshihiro Ishida, Masako Kataoka, Takaki Sakurai, Takayuki Yagi, Kenji Kabashima, Shigehiko Suzuki, Kaori Togashi, Tsuyoshi Shiina, Masakazu Toi

**Affiliations:** 1 Department of Breast Surgery, Graduate School of Medicine, Kyoto University, Kyoto, Japan; 2 Japan Science and Technology Agency, ImPACT Program, Cabinet Office, Kyoto, Japan; 3 Department of Diagnostic Imaging and Nuclear Medicine, Graduate School of Medicine, Kyoto University, Kyoto, Japan; 4 Medical Imaging Development Center, Canon Inc., Kyoto, Japan; 5 Department of Plastic and Reconstructive Surgery, Graduate School of Medicine, Kyoto University, Kyoto, Japan; 6 Department of Dermatology, Graduate School of Medicine, Kyoto University, Kyoto, Japan; 7 Department of Diagnostic Pathology, Graduate School of Medicine, Kyoto University, Kyoto, Japan; 8 Department of Human Health Science, Graduate School of Medicine, Kyoto University, Kyoto, Japan

## Abstract

Photoacoustic (PA) imaging (PAI) has been shown to be a promising tool for non-invasive blood vessel imaging. A PAI system comprising a hemispherical detector array (HDA) has been reported previously as a method providing high morphological reproducibility. However, further improvements in diagnostic capability will require improving the image quality of PAI and fusing functional and morphological imaging. Our newly developed PAI system prototype not only enhances the PA image resolution but also acquires ultrasonic (US) B-mode images at continuous positions in the same coordinate axes. In addition, the pulse-to-pulse alternating laser irradiation shortens the measurement time difference between two wavelengths. We scanned extremities and breasts in an imaging region 140 mm in diameter and obtained 3D-PA images of fine blood vessels, including arterioles and venules. We could estimate whether a vessel was an artery or a vein by using the S-factor obtained from the PA images at two wavelengths, which corresponds approximately to the haemoglobin oxygen saturation. Furthermore, we observed tumour-related blood vessels around breast tumours with unprecedented resolution. In the future, clinical studies with our new PAI system will help to elucidate various mechanisms of vascular-associated diseases and events.

## Introduction

A blood vessel is a passageway for blood and plays a vital role in transporting various components, such as oxygen and nutrients, to each organ of the body. Ailments such as diabetes, cardiovascular disease and collagen diseases, such as rheumatism, involve injury to the peripheral blood vessels. Malignancies such as breast cancer instigate the formation of abnormal blood vessels inside and around tumours by inducing angiogenesis^1,2^. In addition, when performing plastic surgery to recover tissue that has been lost by surgical removal, as in cancer, it is imperative to transplant not only tissues but also blood vessels from the donor to nourish the transplanted tissue. Thus, it is essential to elucidate the form of blood vessels and arteriovenous veins for disease diagnosis and treatment.

The current vascular imaging method used in the medical field requires the use of a contrast agent, and obtaining an image requires X-ray exposure or an expensive contrast magnetic resonance imaging (MRI) examination. In contrast, blood flow imaging in the B-mode is feasible even with a Doppler imager mounted on an ultrasonic (US) diagnostic apparatus and requires no contrast medium nor invasive procedures; however, its resolution is limited.

Photoacoustic (PA) tomography (PAT) is an imaging method that utilises the light absorption of haemoglobin for non-invasive blood vessel imaging^3^. Previous clinical studies have actively investigated the impact of PAT on humans for application in the medical field^4–14^. Instrument configurations in which the linear probe used in a US diagnostic apparatus is provided with a light source have the advantage of enabling the rapid construction of a PA system^14,15^. However, obtaining high-quality images is challenging because of the ‘limited view problem’ of PA phenomena^16^. To resolve this problem, a PAT system comprising a hemispherical detector array (HDA) surrounding an object to be measured with a solid angle wider than that of a linear probe has been reported previously^17–19^. We developed a novel prototype (PAI-04) with enhanced specifications to realise large-area, high-resolution blood vessel analysis. Hence, this study aims to outline the device configuration of our novel PA imaging system prototype, presents examples of *in vivo* images obtained using this device and elucidates its possible clinical applications.

## Results

**Figure 1:**
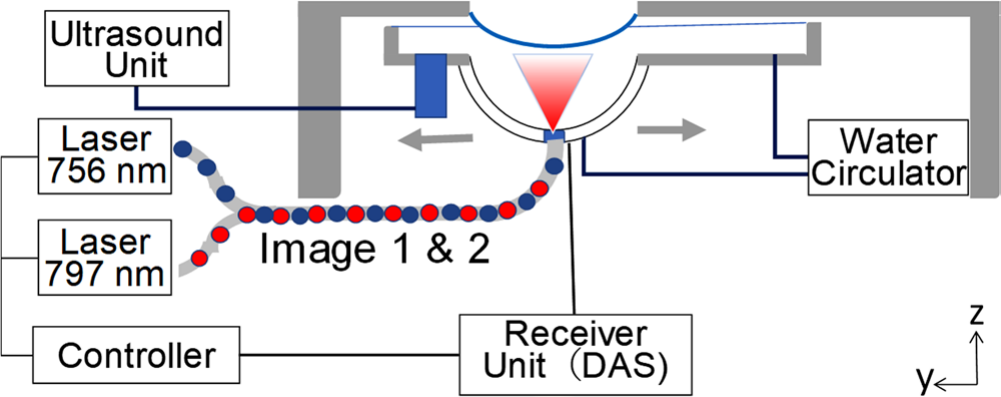
Schematic illustration showing the PAI-04 system configuration and the alternating irradiation sequence (pulse-to-pulse wavelength switching) adopted in this study. The PA controller controls the laser oscillation and PA wave reception. In this apparatus, laser beams of two different wavelengths are used for alternating irradiation. The generated PA wave is received by the hemispherical probe array (HDA) and sent to the data acquisition system (DAS). In addition to the PA controller, another unit controls ultrasonic (US) transmission and reception. Data received by the US transducer are sent to the US unit. The US transducer and the HDA are integrated as a transducer module, and the entire system is configured to move simultaneously during scanning. The space between the holding cup and the transducer module is filled with circulating water. Water for acoustic matching with the test object is poured into the holding cup.

**Figure 2:**
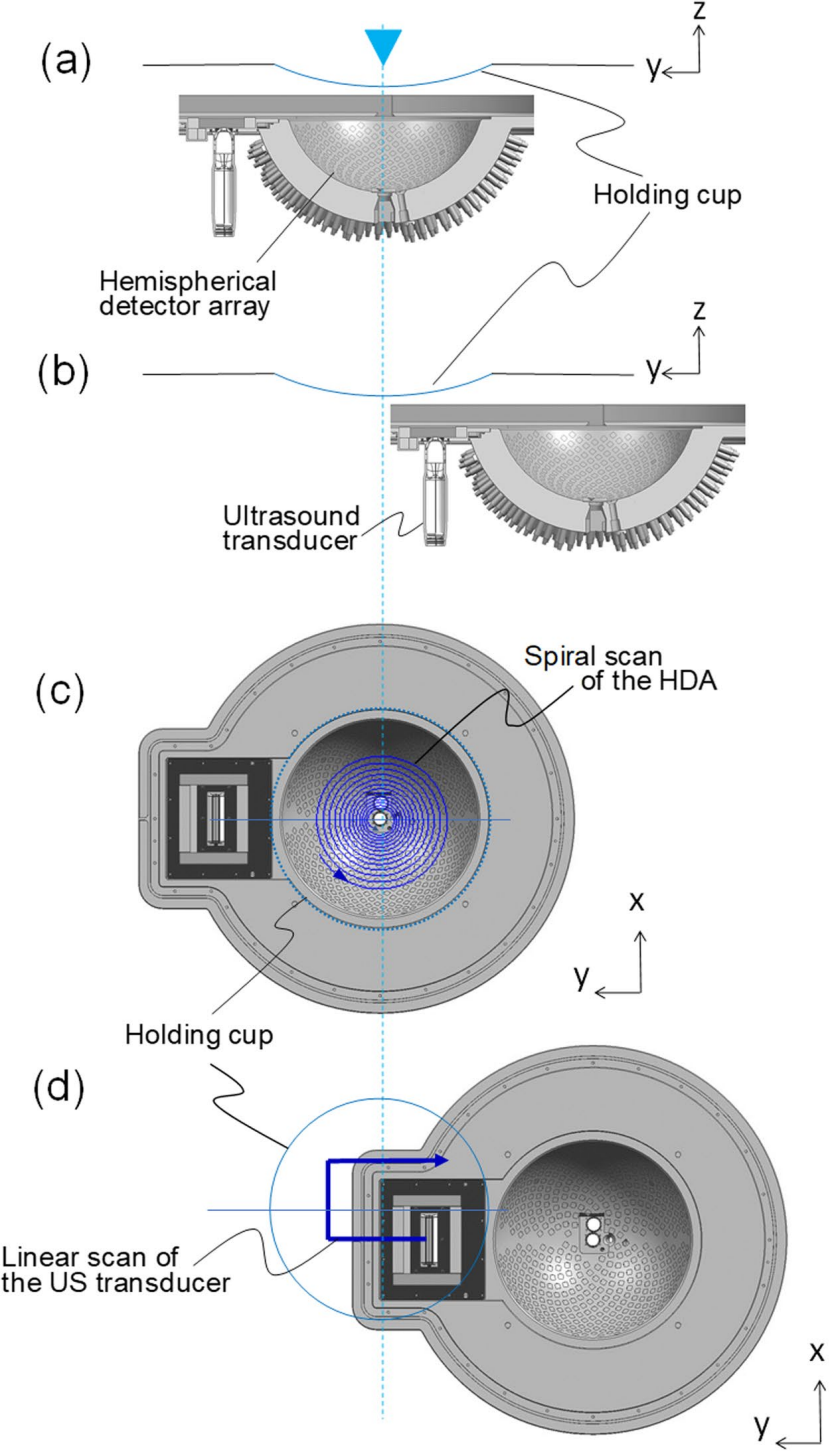
Schematic illustration showing the positional correlation between the breast-holding cup and the HDA. (**a**) A cross-sectional view of the HDA and breast-holding cup cut at the centre during the PA measurement. (**b**) A cross-sectional view of the HDA and breast-holding cup cut at the centre during the US measurement. (**c**) A schematic illustration of the top view showing a scan sequence during the PA measurement. The HDA scans in a spiral shape with the central point of the holding cup as a rotation axis to acquire a PA image in a wide range. (**d**) A schematic illustration of the top view showing a scan sequence of the US measurement after the PA measurement. The US transducer moves under the holding cup and a transducer module and scans in the upward direction from the linear US transducer. When the scanning in the upward direction is completed, the next row is scanned by moving by one transducer width to acquire a wide range of US images.

The elementary design of PAI-04 inherited the design of the conventional prototype (PAI-03)^9,10,19^ equipped with an HDA receiving PA signals. We inserted a test object into the holding cup on the imaging table and performed a scan (Fig. 1; Fig. S1a–c). When imaging was required around the trunk, such as for the breast or thigh, subjects were asked to lie in the prone position on the scanning table. For scanning of the hand, subjects sat beside the table and placed their palm into the holding cup. Then, we scanned the laser light and the HDA helically around the holding cup in a horizontal plane and measured the subjects with a wide angle (Fig. 2a,c). The measurement time was approximately 2 min for an imaging region with a diameter of 140 mm.

In this prototype, we changed the central frequency of the PA signal detector (Fig. S2a–d) from 2 MHz, used in our previous PAI-03, to 4 MHz. Owing to the phantom experiment, the spatial resolution improved from 0.5 mm to 0.27 mm. In our previous study^9^, when measuring images using two wavelengths of 756 nm and 797 nm, we performed the second wavelength scan by switching the wavelength after completing the scan using the first wavelength (Fig. S3a–d). In this study, we adopted alternating light irradiation^20^ with pulse-to-pulse wavelength switching in the PAI-04 (Fig. 1). Another key difference is the simultaneous acquisition of US echo images^14,15,21,22^. We placed a dedicated 256-channel linear transducer, equivalent to that used in a typical clinical setting, beside the HDA for US echo imaging. After performing a spiral scan of the HDA to obtain the PA signal (Fig. 2a,c), we moved the US probe under the subjects and scanned automatically (Fig. 2b,d). The three-dimensional US (3D-US) image acquisition time was approximately 1 min. Then, we reconstructed 3D-US images by stacking B-mode images acquired in a broad range. The 3D-US image acquisition time was approximately 1 min. Furthermore, the data acquisition area of the 3D-US in the *x*–*y* plane comprised a square inscribed in a circle with a diameter of 140 mm.

We evaluated the haemoglobin oxygen saturation (SO_2_) using an approximate parameter calculated from measurements obtained at two wavelengths, which we termed the S-factor. Background optical coefficients were measured beforehand for scanning mammary glands, and we attempted a numerical analysis of the S-factor. Notably, we did not measure the background optical coefficients for other tissues for anatomical reasons. In regions where the background was considered similar, such as for adjacent blood vessels, we projected whether the correlation between the S-factors along the two vessels was consistent with the correlation between their real SO_2_ values and compared the relative values. We acquired a 3D-US image to measure a breast tumour and extracted and stacked the hypoechoic area as a volume image. In addition, the tumour lesion was identified by a breast physician familiar with mammary US imaging. Then, segmentation processing was performed on the basis of the US image intensity. Furthermore, we applied the graph cut method^23,24^ to extract the tumour area.

**Figure 3:**
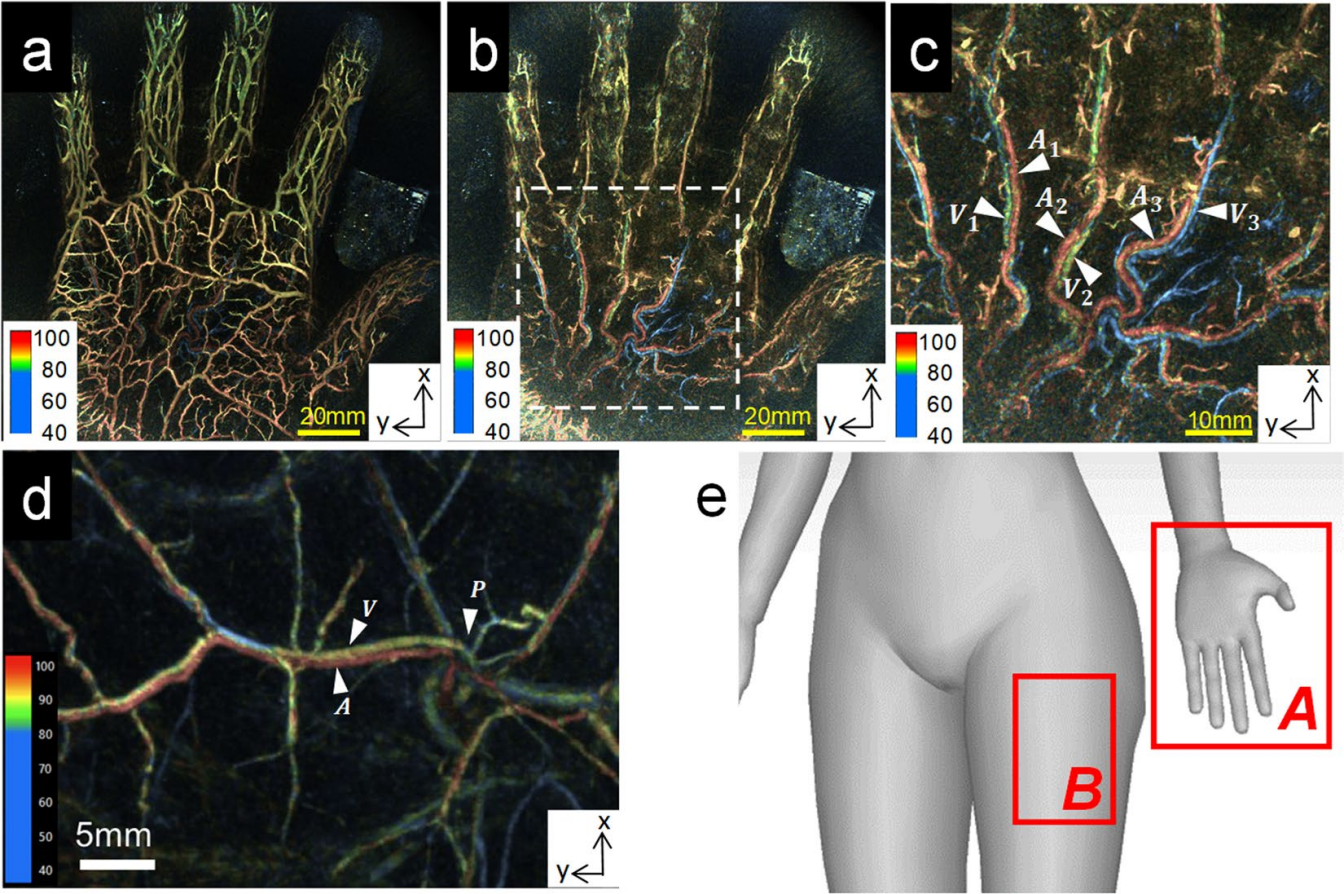
Examples of palm and thigh PA images obtained from healthy subjects *in vivo*. (**a–c**) Examples of palm PA images obtained using an alternate irradiation sequence. No body motion correction was performed. (**a**) The maximum intensity projection (MIP) image of the whole palm. (**b**) An image after deletion of the subcutaneous veins from the whole palm image. (**c**) An enlarged image of the region of (**b**) indicated by the white dashed line shows the common palmar digital arteries. In Fig. 2c, the blood vessels anatomically determined to be the common palmar digital arteries are designated A1–3, and the veins accompanying them are V1–3. The assignment of these arteries and veins corresponded to the blood vessel colour of the S-factor image (i.e., the magnitude relationship of the S-factor). (**d**) An example PAT image obtained in an anterolateral thigh. A stem portion of perforator (P) vessels and a bundle representing an artery (A) and a vein (V) were observed. (**e**) Schematic illustration of the measured tissue in the body of a subject.

Figure 3a–d present examples of image acquisition in the palm (Fig. 3a–c; Fig. S3a–d) and thigh (Fig. 3d) of a healthy subject. Figure 3e shows the scanned position in the living body. We confirmed the effect of alternating irradiation using the image of the palm (Fig. 3a–c; Fig. S3a–d). The S-factor colour of the common palmar digital arteries designated by A1–3 in Fig. 3c (by alternating laser irradiation) was uniform, and two accompanying vessels ran parallel to one another. Conversely, the colour was not uniform in Fig. S3d (conventional sequential irradiation), and the difference between the artery and the vein was not easily discerned. In addition, arteries and veins could be clearly distinguished by nuances in colour in the S-factor image obtained using alternating irradiation in the PAI-04. Figure 3d presents an image of an anterolateral thigh. The correlation between the locations of perforators (P) identified by PA imaging compared with conventional US exhibited discrepancies of <10 mm^11^. In addition, in PA images of bundles of blood vessels near the stem part, the image features comprising the vessel presumed to be the artery (A) and the vessel presumed to be the vein (V) from the image matched the anatomy of the perforators.

**Figure 4:**
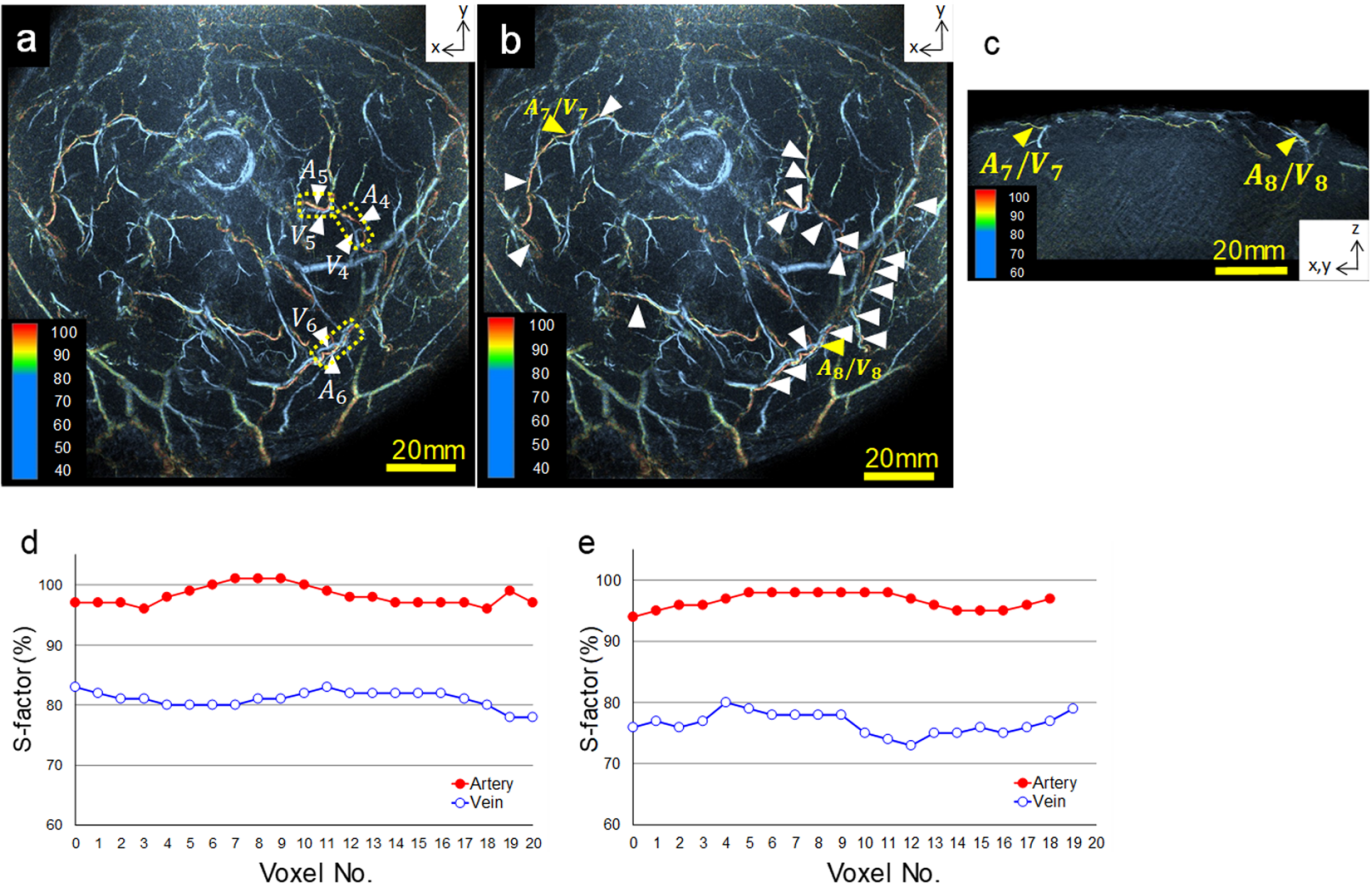
Examples of breast PA images obtained from healthy subjects *in vivo*. (**a**) Three measured positions for the reproducibility evaluation of the S-factor in the breast. The S-factors of three adjacent vessels (A4–6, V4–6) are shown. The measured ranges of the S-factor are indicated by the yellow dotted lines. (**b**) Twenty-three measured positions for evaluating the variation in the S-factor values. (**c**) Examples of the S-factor evaluation of two sets of adjacent vessels (A7/V7 and A8/V8) indicated by a yellow arrowhead in Fig. 3b. The S-factor value in a voxel along a vessel was defined as the average value of the signal at a distance within 2 voxels from the centre of the voxel with a sufficient PA signal intensity to omit background signals. Consequently, the S-factor value calculated at each point of a vessel fluctuated, although the range was very small.

Figure 4a–c present images of a breast. As mentioned earlier, as the background optical coefficients of subjects were measured, we attempted to quantify the S-factor. In addition, the demarcation of arteries and veins by the colour tone (S-factor image) corresponded to the determination of vessels by the anatomical findings. We calculated the S-factors along three different adjacent vessels (Fig. 4a) and performed three measurements to evaluate the reproducibility. Consequently, the coefficient of variation (CV%) of the S-factor of the arteries and veins was <2% (Fig. S4). Then, we evaluated 23 adjacent arteriovenous S-factors (Fig. 4b). A significant difference was observed (*P* = 9.93518E-19) between the S-factor values of the arteries and veins (Table S1), and we considered these absolute values to be reasonable results^25^. Figure 4d,e present examples of the results of quantifying the S-factors of arteries and veins and show the S-factor values for each voxel around A7/V7 and A8/V8, as shown in Fig. 4b,c, respectively. Thus, although the S-factor values evaluated at each point of the blood vessel varied, the range was considered small enough to enable determination of whether they were arteries or veins.

**Figure 5:**
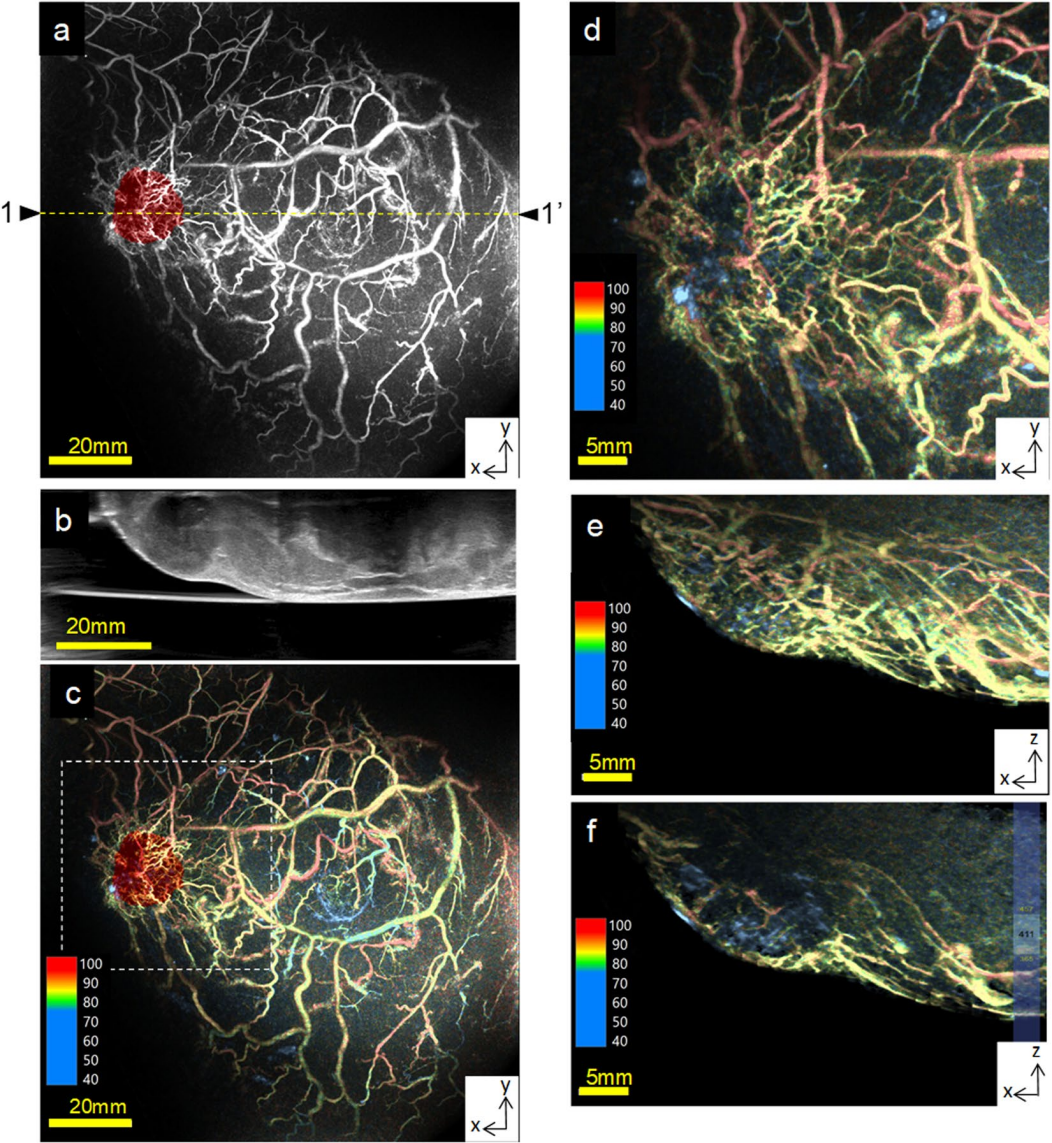
Examples of images obtained from a breast cancer patient *in vivo*. (**a**) A fusion image of the PA image taken at 797 nm and 3D-US images. Hypoechoic portions of the US were extracted based on the US signal intensity and reconstructed into a 3D volume by stacking the continuous B-mode images. The cubed US data were coloured red. Several clustered fine blood vessels were observed in the vicinity of the tumour. (**b**) A US B-mode image of the dotted-line portion indicated by 1–1′ in (**a**). A hypoechoic region is visible and is indicated by the yellow arrowhead. (**c**) A fusion image of the S-factor and 3D-US images (red colour). The nipple at the centre of this image is coloured light blue because the absorption spectrum of melanin in the nipple and the spectrum of low oxygen saturation are almost the same. (**d**) An S-factor image of the same region with enlargement of the tumour vicinity but without fusion with the US tumour image. (**e**) The entire MIP image in the axial direction of the S-factor around the lesion. (**f**) The slab MIP image at the centre of the lesion corresponding to (**e**).

Figure 5a–f show examples of the results obtained for a breast cancer lesion. Figure 5a presents a fusion image of the PA obtained at 797 nm and the 3D-US images (red colour). Notably, fine blood vessels surrounding the tumour are clearly visible. Figure 5b presents a US echo B-mode image of a tumour region. We observed a hypoechoic lesion suggesting a breast tumour in the region, as indicated by an arrow. Figure 5c presents a fusion image of the S-factor and 3D-US images (red colour), and Fig. S5 presents the S-factor and the original image of US C-mode around the tumour. Figure 5d presents the S-factor image of the same region with enlargement of the tumour vicinity and without fusion with the US tumour image. Of note, the arterioles and venules show clustering. In addition, yellow- and green-coloured fine vessels have S-factors of approximately 80–90%, suggesting veins. Figure 5e,f show axial images of the S-factor at the edge and the central portion of the tumour, respectively. In this case, a few blood vessels surrounding or inside the tumour exhibited the oxygen saturation of arteries, and several blood vessels with S-factors corresponding to veins surrounded the tumour.

In addition, we observed some scattered hypoxic points, which most likely represented intratumoural bleeding. The measured diameters of the spots were 700 to 900 µm, as indicated by the yellow arrows in Fig. S6a. This patient underwent tumour resection after PAI observation. The pathology specialist microscopically observed a haematoxylin and eosin (H&E)-stained specimen of the tumour tissue and a CD31-stained specimen of the same area (Fig. S6b–g), which revealed a fibrous core at the centre of the tumour and indicated the decline in endothelial cells of the blood vessels. The central portion of the tumour, where the PA image displayed low and variable SO_2_, might be avascularised by necrosis and fibrosis.

## Discussion

The PAI-04 enabled the visualisation of high-resolution images of arterioles and venules. The improvement in the resolution over that of the conventional prototype could be attributed to the enhanced central frequency. However, it is imperative to carefully consider the use of higher frequency depending on the object measured and the desired application because when the penetration depth decreases because of US attenuation or when the relative bandwidth of the detector is limited, the concern arises that the receivable low-frequency signal is limited and that image quality with good morphological reproducibility cannot be obtained. In addition, with alternating laser irradiation to enhance the S-factor reproducibility, blood vessels anatomically identified as arteries or veins were depicted as relative colours in the S-factor image. Thus, even if the light fluence is unknown, the magnitude relationship of the oxygen saturation can be clinically assessed, demonstrating the validity of the hypothesis that the S-factor reflects the relative SO_2_ value in the neighbouring region.

In thigh imaging, the PA imaging contributed to visualising not only the branching morphology of perforators but also the arteries and veins in the subcutaneous layer. Precise information on subcutaneous vessels will enable surgeons to plan safer procedures for anterolateral thigh flap surgery. In breast cancer imaging, small tumour-related blood vessels could be visualised. Although *in vivo* imaging of tumour-related blood vessels has been reported at the xenograft level^2^, to the best of our knowledge, this study is the first to report this level of detail for tumour-related blood vessels in human cancer tissue. The imaging of fine vessels could be attributed to the fact that the tumour in this study was located near the body surface, with a depth <10 mm from the skin. In the future, we intend to extend the imaging technology for tumour-related blood vessels to deeper positions by enhancing the sensor sensitivity and optimising the laser irradiation method. Because breasts are relatively easy to deform, developing suitable breast positioning to obtain clear PA images using this device is a major goal. We will soon report the results of systemic therapy based on changes in the S-factor in tumour-related blood vessels.

The 3D-US image was markedly effective in identifying the tumour position. By adopting a combination with a US diagnostic system using a linear probe, it was relatively easy to compare the B-mode image usually obtained for diagnosis at the clinical site with the PA image. Conversely, when combining a separate system with a linear probe, a scanning time difference of approximately 2 min occurs, as the US probe is moved after PA imaging. If the subject moves during this period, there is no way to correct for the discrepancy. Thus, physicians should monitor patient movement during the scanning period and carefully assess the correlation between morphology and metabolic function. More reliable data is expected to be obtained from future prototypes by adopting a system configuration that performs the reception of PA signals and US transmission/reception simultaneously^7,14,15,21,22^. In addition, new clinical knowledge will be generated by improving the device and advancing the analytical methods. As our device can be used to comprehensively analyse the vessel branching structure and oxygen metabolism of the human body, it could contribute to various biological assessments *in vivo* and provide clinically important findings. For example, it might be feasible to observe the normalisation of blood vessels in response to drug treatment non-invasively. Perhaps the effect of drug treatment could be evaluated at an early stage, which could contribute to individualised medicine. Furthermore, analysis combining PAI and conventional modalities promises to create a new paradigm for disease diagnosis.

In conclusion, we have developed a high-resolution PAT system prototype capable of laser irradiation with pulse-to-pulse wavelength switching that could obtain 3D-PA and 3D-US images. In addition, arterioles and venules could be imaged and distinguished using S-factor images. In the future, clinical studies with a high-resolution PAI-04 system will help to elucidate various aspects of vascular-associated diseases and events.

## Methods

### Device configuration

We used the PAI-04 system with an HDA (Canon Inc., Tokyo, Japan). We placed the tissue of the subjects intended for measurement in the breast-holding cup on an exam table (Figs 1 and S1). In this study, we referred to the *x*–*y* Cartesian plane as the area parallel to the horizontal plane or parallel to the plane of the exam table surface. The *x*- and *y*-axes were the short and long axis of the table, respectively, and the *z*-axis was the vertical axis. We used one laser system per wavelength to emit laser light with a very large pulse. Although a prior study^26,27^ reported the advantage of spectrum analysis employing multiple wavelengths, we adopted a PAT system with two wavelengths to aim for future practical applications with a straightforward configuration, as in our conventional system^7,9^. In addition, we verified whether the oxygen saturation of the blood in the living body could be measured using this two-wavelength system. We used two Ti:Sa lasers optically pumped using a Q-switched Nd:YAG laser, which can select wavelengths from 700 to 900 nm. We selected two wavelengths, 756 and 797 nm, to construct an improved haemoglobin saturation distribution image. While 756 nm is the local maximum of the absorption coefficient of deoxidised haemoglobin, 797 nm is the isosbestic point of oxyhaemoglobin and deoxidised haemoglobin.

In this study, the laser lights of these two wavelengths alternated in every irradiation pulse along the *z*-axis. Notably, the maximum permissible exposure values recommended by the American National Standards Institute for 756 and 797 nm are 12.9 and 15.6 mJ/cm^2^, respectively. The energy of each laser used in the PAM-04 was 89 and 89 mJ/pulse for 756 and 797 nm, respectively. We set the light energy density for 756 and 797 nm to maximum values of 10.0 and 11.9 mJ/cm^2^, respectively. Furthermore, an 8-mm-diameter pulsed laser beam was fed through a light fibre that directed the laser beam upward along the vertical axis of the transducer array as the HDA was scanned. A diverging lens placed at the base of the HDA spread the light conically to a diameter of 54 mm at the surface of the holding cup. Each laser light source corresponding to each wavelength was emitted sequentially at 10 Hz. Thus, the pulse sequence of (1) pulse laser irradiation at 797 nm, (2) irradiation at 756 nm after 50 ms, and (3) irradiation at 797 nm after 50 ms was repeated.

We used an internally manufactured broadband capacitive micromachined US transducer (CMUT) as a receive-only transducer element to detect the PA signal. In addition, we constructed the detector array for PA signal detection to correspond to the HDA, and its appearance was almost the same as in our prior study (PAI-03)^9,19^. Overall, 500 spirally arrayed CMUT elements were present on the HDA; each element measured 1.5 mm in diameter, and the elements were set at approximately 10-mm intervals. The central frequency and fractional bandwidth of the CMUT element were 4 MHz and >100%, respectively. Notably, the average conversion efficiency was 63 mV/kPa at 4 MHz, and its standard deviation was 2.5 mV/kPa over 500 elements. Furthermore, the noise-equivalent pressure (NEP) of this system was 0.5 Pa at the root-mean-square value without signal averaging. We examined the NEP measurement with a low-pass filter with a cut-off frequency of 15 MHz.

The key difference between PAI-04 and PAI-03 was the addition of US echo imaging. PAI-04 used a linear transducer array as a dedicated transducer for US echo imaging because the CMUT device used in the HDA was designed as a receive-only device. In addition, the 256-channel linear US probe was placed adjacent to the HDA. While the US beam was emitted along the *z*-axis, the electronic scanning direction of the linear probe was along the *x*-axis. Using probes of the same shape as in routine clinical practice facilitated the interpretation of US images. Water was used between the HDA and the breast-holding cup for acoustic matching and was supplied from a water circulation device set at a predetermined temperature. A drain outlet was provided on the bottom of the HDA. The subjects were inserted into the body-temperature water and scanned. For breasts, the affected breast was scanned first, followed by the contralateral side. Lesions were identified primarily using the other hand-held US machine.

### Data acquisition

The HDA was scanned helically in the *x*–*y* plane with the same trajectory as described previously^19^ to obtain a PA image of a large area of the subject. It did not move in the *z*-axis direction. We acquired a PA signal in a range with a diameter of 140 mm using a spiral scan. The received PA signal data were amplified by a variable gain amplifier (VGA) capable of settings up to 10–42 dB and converted to digital data with a 40 MHz, 12-bit AD converter. We primarily set the amplification rate to 30 dB in this clinical study.

For a 140-mm-diameter area, the measurement time for a PA signal was approximately 2 min. To obtain a US image after PA scanning, we obtained US B-mode images at continuous positions by moving the US linear probe under the subjects and scanning along the *y*-axis. Then, a 3D-US image was obtained by stacking the B-mode images. The probe was shifted along the *x*-axis after the one-line scan and the same scan was performed along the *y*-axis to measure a range greater than the probe width in the *x*-axis direction. Then, the scanned US data were combined after the measurement and converted into a 3D-US image. The measurement time for a US image was approximately 1 min for a 100-mm-square area. When examining patients with breast cancer, the state of the tumour was confirmed beforehand with a clinical US apparatus, and a breast surgeon selected an appropriate scan area based on the anatomy of the patient’s breast and the extent of the tumour spread. In cases other than breast cancer, we did not obtain 3D-US echo images and evaluated only PA images.

### Imaging method

We used universal back-projection (UBP)^28^ for the PA image reconstruction. The reconstructed voxel size was typically 0.0625 or 0.125 mm and was selected depending on the size of the object and the type of analysis. We created an image for each laser shot and constructed the whole image by combining these images (Supplemental Video 4). Since the water temperature differed between the water above and below the holding cup, we performed image reconstruction by evaluating the sound velocity, which was categorised into two layers based on the difference in the sound velocity because of the temperature difference^29^.

When imaging a breast, we measured the background optical parameter beforehand with the TRS-20 (Hamamatsu Photonics K.K., Hamamatsu, Japan)^30^ and obtained an absorption coefficient distribution by roughly evaluating the light fluence distribution. In this study, we hypothesised that the absolute value of SO_2_ could be approximately evaluated. Conversely, when tissues other than the breast were measured (e.g., the palm), the derivation of the background optical constant was challenging because tissues, such as muscles, tendons and bones, were intertwined with each other. Thus, we performed S-factor analysis based only on the measured initial sound pressure distribution without evaluating the absorption coefficient distribution. Even in that case, the magnitude correlation between the true oxygen saturation and the measured oxygen saturation, which we called the S-factor, was not reversed. Hence, we did not consider the absolute value of oxygen saturation as a subject of discussion and instead considered it as a relative value. The calculation of the S-factor and its validity for relative assessment are described later. In addition, we did not evaluate the light fluence except in the breast evaluation and obtained images using only the measured initial acoustic pressure distribution, with the light fluence ratio assumed to be 1; this calculation or assumption was expressed in the S-factor image. For S-factor images in this study, the S-factor value and the absorption coefficient at 797 nm were indicated by the hue and intensity, respectively.

In this study, as in our previous study, we used two types of body motion correction methods for image reconstruction^9^. The first was a correction for body motion between different laser pulses (p-BMC), and the second was a correction between two different laser wavelengths (w-BMC) for the S-factor image. We used p-BMC and not w-BMC in this study, except for the comparative experiments (Fig. S3). We selected p-BMC because the movement of the living body at rest during the 50-ms pulse interval was assumed to be negligible, even in the case of the high-resolution PA image, where the resolution was several hundred micrometres. In addition, when scanning the breast, a 3D-US image was acquired using the scanning sequence (Fig. 2c). We constructed 3D-US images by scanning the ultrasonic linear array probe along the elevation direction and creating an image in which successive B-mode images were stacked.

The coordinates of the constructed 3D-US data were set to coincide with the coordinates of the PA image so that two could be overlaid on an image viewer^31^. We extracted the low-echo area of the US based on the intensity information from the US image to compare and analyse the obtained 3D-US image and PA image. Regarding mammary tumours, physicians familiar with the US diagnosis of breast cancer manually assigned a hypoechoic region inside the tumour. Then, by applying the graph cut method^23,24^ with the hypoechoic region as the seed point, we extracted the curved boundary surface between the tumour and the nontumour region to derive the 3D structure. After binary processing with the extracted tumour area set to 1 and other areas set to 0, we fused the US image of the tumour volume alone in the red image with the PA image. For the S-factor, colour imaging was adopted in which the value of the S-factor corresponded to the hue and the image intensity value to the absorption coefficient at 797 nm, which provided an image approximating the weight distribution of the total haemoglobin. After obtaining the PA image, we deleted the data near the subcutaneous tissue by image processing as necessary. Next, we applied a cloth simulation method to obtain information on the body surface^31,32^. Furthermore, the volume data were trimmed from the body surface position to an arbitrary depth to simplify the image interpretation of deeper tissues.

### Error between the S-factor and the oxygen saturation

We adopted the following method to evaluate the haemoglobin oxygen saturation using a two-wavelength light source. However, quantitative PA imaging is a major problem that requires a solution. In particular, quantitatively and precisely evaluating the absolute value of the absorption coefficient (*μ*_a_) is difficult because of the limited view problem^7^ along with difficulty in estimating the light fluence. If the two *μ*_a_ values of the absorber with wavelengths *λ*_1_ and *λ*_2_ are obtained correctly, the Hb saturation value (SO_2_) can be derived from the following equation:

1 $${{\rm{SO}}}_{2}=\frac{[{{\rm{HbO}}}_{2}]}{[{{\rm{HbO}}}_{2}]+[{\rm{Hb}}]}=\frac{\frac{{\mu }_{a}^{{\lambda }_{2}}(r)}{{\mu }_{a}^{{\lambda }_{1}}(r)}\cdot {\varepsilon }_{{\rm{Hb}}}^{{\lambda }_{1}}-{\varepsilon }_{{\rm{Hb}}}^{{\lambda }_{2}}}{{\varepsilon }_{{\rm{\Delta }}\mathrm{Hb}}^{{\lambda }_{2}}-\frac{{\mu }_{a}^{{\lambda }_{2}}(r)}{{\mu }_{a}^{{\lambda }_{1}}(r)}\cdot {\varepsilon }_{{\rm{\Delta }}\mathrm{Hb}}^{{\lambda }_{1}}}$$

where *λ*_1_ and *λ*_2_ represent wavelengths of 756 and 797 nm, respectively; *r* is the position to be calculated; *ε*_Hb_ is the molar extinction coefficient of deoxy-Hb; and *ε*_*Δ*Hb_ is the difference in the molar extinction coefficients between deoxy-Hb and oxy-Hb (HbO_2_). However, in practice, the absolute value of SO_2_ is unreliable because the light fluence affects the absolute SO_2_ value. Hence, we adopted the following method to extract the semiquantitative information *in vivo*.

To evaluate *μ*_a_, Eq. 2 was derived as follows:

2 $${\mu }_{a}^{{\lambda }_{1}}(r)=\frac{{p}_{0}^{{\lambda }_{i}}(r)}{{\rm{\Gamma }}\cdot {\varphi }^{{\lambda }_{i}}(r)}\,,\,i=1,2$$

where *p*_0_, Γ and ϕ are the initial pressure of PA, the Grüneisen parameter and the light fluence, respectively. In addition, *λ*_1_ and *λ*_2_ represent wavelengths of 756 and 797 nm, respectively.

Eq. 1 can be transformed as follows:

3 $${{\rm{SO}}}_{2}(r)=\frac{\frac{{p}_{0}^{{\lambda }_{2}}(r)}{{p}_{0}^{{\lambda }_{1}}(r)}\cdot \frac{{\varphi }^{{\lambda }_{1}}(r)}{{\varphi }^{{\lambda }_{2}}(r)}\cdot {\varepsilon }_{{\rm{Hb}}}^{{\lambda }_{1}}-{\varepsilon }_{{\rm{Hb}}}^{{\lambda }_{2}}}{{\varepsilon }_{{\rm{\Delta }}\mathrm{Hb}}^{{\lambda }_{2}}-\frac{{p}_{0}^{{\lambda }_{2}}(r)}{{p}_{0}^{{\lambda }_{1}}(r)}\cdot \frac{{\varphi }^{{\lambda }_{1}}(r)}{{\varphi }^{{\lambda }_{2}}(r)}\cdot {\varepsilon }_{{\rm{\Delta }}\mathrm{Hb}}^{{\lambda }_{1}}}$$

Here, the correlation between the reconstructed initial pressure ${p}_{{\rm{meas}}}(r)$ and the true initial pressure ${p}_{0}(r)$ can be expressed as follows:

4 $${p}_{{\rm{meas}}}^{{\lambda }_{i}}(r)={p}_{0}^{{\lambda }_{i}}(r)\cdot {U}^{{\lambda }_{i}}(r)+{A}^{{\lambda }_{i}}(r),\,\,i=1,\,2$$

where *U* is a correction term expressing the variation in the acoustic properties accompanying the propagation and reception of PA waves because of acoustic diffusion, acoustic attenuation, the limited view problem (caused by the small receiving angle), the central frequency and the fractional bandwidth of the probe. In addition, the acoustic correction term *U* can be considered to have approximately the same value for both wavelengths except for cases where the shape of the initial pressure or the frequency characteristics of the PA signals differ between the two wavelengths because of the extreme wavelength dependence of light absorbance. *A* is a term expressing noise and artefacts. We used the term S-factor (*S*_*f*_) instead of oxygen saturation (SO_2_) for the approximation to avoid misunderstanding. Based on this information, Eq. 3 can be approximated as follows:

5 $${S}_{f}(r)=\frac{M(r)\cdot {\varepsilon }_{{\rm{Hb}}}^{{\lambda }_{1}}-{\varepsilon }_{{\rm{Hb}}}^{{\lambda }_{2}}}{{\varepsilon }_{{\rm{\Delta }}\mathrm{Hb}}^{{\lambda }_{2}}-M(r)\cdot {\varepsilon }_{{\rm{\Delta }}\mathrm{Hb}}^{{\lambda }_{1}}}$$

6 $$M(r)=\frac{{p}_{{\rm{meas}}}^{{\lambda }_{2}}(r)-{A}^{{\lambda }_{2}}(r)}{{p}_{{\rm{meas}}}^{{\lambda }_{1}}(r)-{A}^{{\lambda }_{1}}(r)}\cdot \frac{{\varphi }^{{\lambda }_{1}}(r)}{{\varphi }^{{\lambda }_{2}}(r)}$$

In Eq. 6, the initial acoustic pressure at each wavelength can be evaluated by the image reconstruction of UBP using the signal obtained by the PAT system. Thus, the S-factor accuracy depends on estimation of the ratio between the light fluence of the two wavelengths in *M (r)* and the amount of noise and artefacts. When the ratio between the light fluence of the two wavelengths can be regarded as uniform, the following equation is derived:

7 $$\frac{\partial ({S}_{f})}{\partial ({{\rm{SO}}}_{2})}=\frac{\partial ({S}_{f})}{\partial (\frac{{\varphi }^{{\lambda }_{1}}(r)}{{\varphi }^{{\lambda }_{2}}(r)})}\cdot \frac{1}{\frac{\partial ({{\rm{SO}}}_{2})}{\partial (\frac{{\varphi }^{{\lambda }_{1}}(r)}{{\varphi }^{{\lambda }_{2}}(r)})}}$$

8 $$={\{\frac{\frac{{\varphi }^{{\lambda }_{1}}(r)}{{\varphi }^{{\lambda }_{2}}(r)}\cdot {\varepsilon }_{{\rm{\Delta }}\mathrm{Hb}}^{{\lambda }_{2}}-\frac{{p}_{0}^{{\lambda }_{2}}(r)}{{p}_{0}^{{\lambda }_{1}}(r)}\cdot {\varepsilon }_{{\rm{\Delta }}\mathrm{Hb}}^{{\lambda }_{1}}}{\frac{{\varphi }^{{\lambda }_{1}}(r)}{{\varphi }^{{\lambda }_{2}}(r)}\cdot {\varepsilon }_{{\rm{\Delta }}\mathrm{Hb}}^{{\lambda }_{2}}-\frac{{p}_{{\rm{meas}}}^{{\lambda }_{2}}(r)-{A}^{{\lambda }_{2}}(r)}{{p}_{{\rm{meas}}}^{{\lambda }_{1}}(r)-{A}^{{\lambda }_{1}}(r)}\cdot {\varepsilon }_{{\rm{\Delta }}\mathrm{Hb}}^{{\lambda }_{1}}}\}}^{2}\cdot \frac{{p}_{0}^{{\lambda }_{1}}(r)}{{p}_{0}^{{\lambda }_{2}}(r)}\cdot \frac{{p}_{{\rm{meas}}}^{{\lambda }_{2}}(r)-{A}^{{\lambda }_{2}}(r)}{{p}_{{\rm{meas}}}^{{\lambda }_{1}}(r)-{A}^{{\lambda }_{1}}(r)}$$

9 $$={\{\frac{\frac{{\varphi }^{{\lambda }_{1}}(r)}{{\varphi }^{{\lambda }_{2}}(r)}\cdot {\varepsilon }_{{\rm{\Delta }}\mathrm{Hb}}^{{\lambda }_{2}}-\frac{{p}_{0}^{{\lambda }_{2}}(r)}{{p}_{0}^{{\lambda }_{1}}(r)}\cdot {\varepsilon }_{{\rm{\Delta }}\mathrm{Hb}}^{{\lambda }_{1}}}{\frac{{\varphi }^{{\lambda }_{1}}(r)}{{\varphi }^{{\lambda }_{2}}(r)}\cdot {\varepsilon }_{{\rm{\Delta }}\mathrm{Hb}}^{{\lambda }_{2}}-\frac{{p}_{{\rm{meas}}}^{{\lambda }_{2}}(r)-{A}^{{\lambda }_{2}}(r)}{{p}_{{\rm{meas}}}^{{\lambda }_{1}}(r)-{A}^{{\lambda }_{1}}(r)}\cdot {\varepsilon }_{{\rm{\Delta }}\mathrm{Hb}}^{{\lambda }_{1}}}\}}^{2}\cdot \frac{{p}_{0}^{{\lambda }_{1}}(r)}{{p}_{0}^{{\lambda }_{2}}(r)}\cdot \frac{{A}^{{\lambda }_{2}}(r)}{{A}^{{\lambda }_{1}}(r)}\cdot \frac{{{\rm{SNR}}}^{{\lambda }_{2}}(r)-1}{{{\rm{SNR}}}^{{\lambda }_{1}}(r)-1}$$

In Eq. 9, the first, second and third terms on the right side are always positive. Thus, $\partial ({S}_{f})/\partial ({{\rm{SO}}}_{2})$ can be considered always positive from term 4, at least when the signal-to-noise ratios (SNRs) are >1 for both wavelengths. In other words, when the ratio between the light fluence of the two wavelengths of subjects is constant (i.e., when the tissue can be assumed to be a homogeneous medium), the magnitude relationship of the oxygen saturation of the obtained blood vessel images can be assessed. In contrast, when the ratio of the light fluence of the subjects is not considered constant, even relative comparisons require attention. For example, comparing S-factors is challenging for far-distant blood vessels because the light fluence is altered by the impact of the tissue on the light paths leading to the respective blood vessels. Conversely, when comparing blood vessels in close proximity to each other, such as adjacent arteries and veins, the light fluence reaching the area is assumed to be constant, and the S-factors can be evaluated. Figure S7 presents the error rate of the light fluence ratio and the numerical value of the S-factor evaluated in Eq. 5. The noise component was ignored in this study. The five lines in the graph represent numerical values when the correct oxygen saturation value is 100%, 90%, 80%, 70% and 60%. This graph illustrates the validity of Eq. 9 because the magnitude relation of the S-factor is upheld if there is an error in light fluence. Furthermore, the gradient of the error as the oxygen saturation increases is gradual.

### Data analysis

As mentioned earlier, even if the fluence of light is unknown, the magnitude correlation between the actual SO_2_ and the S-factor of the blood vessel image does not alter. Thus, we attempted a relative assessment of the S-factor in the limb measurement to distinguish between the vein and the artery. In this clinical study, it was not feasible to invasively confirm either an artery or a vein in humans because the premise was non-invasive measurement. Thus, whether a blood vessel was an artery or a vein was estimated depending on the morphological knowledge and the relationship of the haemoglobin oxygen saturation. Morphologically, it is anatomically known that veins accompany arteries; that is, one or two veins and one artery are often adjacent. Furthermore, the haemoglobin oxygen saturation of venous blood is lower than that of arterial blood^25^. In this study, a surgeon presumed either an artery or a vein based on the above knowledge. In the clinical setting, we would expect to visually confirm whether the vessel was an artery or a vein at the time of the skin tissue incision.

Regarding clinical studies for breast cancer diagnosis, the background optical coefficient and the light fluence distribution in the breast were evaluated in advance based on the TRS-20 measurement results described earlier. We verified the validity of the quantitative S-factor value as follows. First, the reproducibility was evaluated. Then, we arbitrarily selected arteries and veins adjacent to each other in a range of at least 10 mm at three different positions in a normal breast and evaluated their average S-factor. After confirming that the condition of the subjects was stable, we performed three measurements and obtained the average value and CV% of the S-factor. Next, we selected 23 sets of adjacent vessels (associated with arteries and veins) capable of tracing over 15 pixels in the same breast. Then, the mean value of the S-factor of the blood vessel in that range was obtained and compared between the arteries and veins. In addition, we assessed whether a significant difference was detected between the arteries and veins using Welch’s *t*-test to detect differences between S-factors. Regarding the validity of the absolute value, we used the average value of a pulse oximeter for the artery. Finally, we validated the vein by considering the average value of the mixed venous blood oxygen saturation measured by a Swan-Ganz catheter^33^ and the average value of the oxygen saturation at the central vein.

Postoperatively, we sectioned excised specimens at 5 mm intervals perpendicular to the longest axis of a specimen and performed pathological analysis on permanent formalin-fixed paraffin-embedded (FFPE) tissues by conventional H&E staining. In addition, we used the anti-CD31 monoclonal antibody JC70A (Dako, Santa Clara, CA 95051) to visualise microvessels by immunohistochemistry at a dilution of 1:50 overnight after heat-induced antigen retrieval in Dako Target Retrieval solution pH 9 for 30 min, according to the manufacturer’s instructions.

### Ethics

This study was approved by the Ethics Committee of the Kyoto University Graduate School of Medicine (UMIN000018893, UMIN000022215 and UMIN000022767), and we obtained written informed consent from all subjects. This study was conducted in accordance with the Declaration of Helsinki. For UMIN000022215, female patients with a breast lesion or lesions who fulfilled all inclusion criteria and who did not meet the exclusion criteria were enrolled. The subjects of the UMIN000018893 and UMIN000022767 studies were healthy volunteers whose palms or thighs were imaged using our PA system. Detailed results of each clinical trial will be reported in a future article.

## Electronic supplementary material

Supplementary Video 1

Supplementary Video 2

Supplementary Video 3

Supplementary Video 4

Supplementary Information
