# Supplementary material for: Visualising peripheral arterioles and venules through high-resolution and large-area photoacoustic imaging
Source: Sci Rep. 2018 Oct 8;8:14930. doi: 10.1038/s41598-018-33255-8 (PMC6175891; doi:10.1038/s41598-018-33255-8)
Supplement: Supplementary file 5 — Supplementary Information [file 41598_2018_33255_MOESM5_ESM.docx]

Visualising peripheral arterioles and venules through high-resolution and large-area photoacoustic imaging

Yoshiaki Matsumoto^1^, Yasufumi Asao^1,2^, Hiroyuki Sekiguchi^3^, Aya Yoshikawa^1^, Tomoko Ishii^1^, Ken-ichi Nagae^4^, Shuichi Kobayashi^4^, Itaru Tsuge^5^, Susumu Saito^5^, Masahiro Takada^1^, Yoshihiro Ishida^6^, Masako Kataoka^3^, Takaki Sakurai^7^, Takayuki Yagi^2^, Kenji Kabashima^6^, Shigehiko Suzuki^5^, Kaori Togashi^3^, Tsuyoshi Shiina^8^ and Masakazu Toi^1^

1 Department of Breast Surgery, Graduate School of Medicine, Kyoto University, Japan

2 Japan Science and Technology Agency, ImPACT Program, Cabinet Office, Japan

3 Department of Diagnostic Imaging and Nuclear Medicine, Graduate School of Medicine, Kyoto University, Japan

4 Medical Imaging Development Center, Canon Inc., Japan

5 Department of Plastic and Reconstructive Surgery, Graduate School of Medicine, Kyoto University, Japan

6 Department of Dermatology, Graduate School of Medicine, Kyoto University, Japan

7 Department of Diagnostic Pathology, Graduate School of Medicine, Kyoto University, Japan

8 Department of Human Health Science, Graduate School of Medicine, Kyoto University, Japan

Yoshiaki Matsumoto and Yasufumi Asao contributed equally to this work.

Correspondence should be addressed to Masakazu Toi (toi@kuhp.kyoto-u.ac.jp)

***Supplementary Figures***

***Supplementary Figure 1***

Photographs of the PAI-04 system as a whole.

(a)

A photograph of the side view.

(b)

A photograph of the foot side.

(c)

A photograph from an oblique top view. The yellow arrow indicates the holding cup. A test object is scanned after proper positioning.

***Supplementary Figure 2***

Figures that describe the hemispherical detector array (HDA) adopted in this article.

A schematic illustration of the transducer module, consisting of an HDA and an ultrasound (US) linear probe, viewed obliquely from the top.

(b)

A schematic illustration of the transducer module viewed from the top.

(c)

Cross-sectional view of the HDA cut at the centre.

(d)

A photograph of a CMUT stick used for the HDA. Five hundred CMUT sticks are inserted into the hemispherical case from the outside and constitute the HDA used for this article.

***Supplementary Figure 3***

Schematic illustration showing a conventional sequential irradiation sequence and the palm PA images obtained using a conventional sequential irradiation sequence.

(a) Schematic illustration showing a conventional sequential irradiation sequence. After obtaining an image at 756 nm, an image was obtained at 797 nm to generate an S-factor image. A body motion correction was performed between the two laser wavelengths (w-BMC).

(b-d) Examples of the palm PA images obtained using a conventional sequential irradiation sequence. (b) A maximum intensity projection (MIP) image of the whole palm. (c) An image after deletion of the subcutaneous veins from the whole palm image. (d) An enlarged image of the part of Figure S3c indicated by the white dashed line, showing the common palmar digital arteries. Although the colour of the S-factor in the common palmar digital arteries indicated by A1-3 in Fig. 2c (alternating irradiation) is uniform and two accompanying veins run parallel to one another, the colour of the S-factor in the common palmar digital arteries in Fig. S3d (conventional sequential irradiation) is not uniform, and the difference between the artery and the vein is not easily recognised.

***Supplementary Figure 4***

Reproducibility of the S-factor measurement. S-factors of an adjacent artery and vein at three different positions (*A_4-6_* and *V_4-6_* in Fig. 3-a) in a normal breast were calculated. A total of three independent measurements were performed, and the average value and the coefficient of variation (CV%) of the S-factor were obtained. The CV% of all points was less than 2%, which showed the high reproducibility of this measurement.

***Supplementary Figure 5***

A fused image of PA and US in the C-mode across the centre of the tumour without performing US tumour region extraction, as shown in Fig. 5c. The greyscale represents the US image, and the colour represents the PA image. Analysis of the relationship between the tumour and the blood vessel is difficult due to the existence of a low-echo region not only in the tumour area but also in the mammary gland layer.

***Supplementary Figure 6***

(a)

An image indicating the measured diameters of the spots showing scattered hypoxic regions in the tumour. Measured spots are indicated by yellow arrows. The minor axes of S1 and S2 were 0.94 mm and 0.77 mm, respectively.

(b)

Microscopic image of the excised tumour after H&E staining, revealing a necrotic area.

(c)

Microscopic image near the section of Fig. S6b of the excised tumour after CD31 immunohistochemistry staining, revealing a necrotic area.

(d)

A schematic illustration showing the necrotic area.

(e)

Microscopic image of the other section a few mm from that in Fig. S6b after H&E staining, revealing a fibrous core.

(f)

Microscopic image near the section of the excised tumour shown in Fig. S6e after CD-31 immunohistochemistry staining, revealing a fibrous core.

(g)

A schematic illustration showing the fibrous area.

***Supplementary Figure 7***

A graph of the numerical value of the S-factor calculated in Equation 5 as a function of the error rate of the light fluence ratio. In this calculation, the noise component was ignored. The five lines in the graph represent the numerical values when the correct SO_2_ values are 100, 90, 80, 70 and 60%, respectively.

***Supplementary Table***

***Supplementary Table 1***

S-factor values of 23 sets of adjacent vessels (Fig. 3b). The mean S-factor values of the blood vessels were compared between the arteries and the veins. The S-factor was significantly different between arteries and veins using Welch’s t-test. Regarding the validity of the absolute value, the average value of the pulse oximeter was used for the artery. The vein was validated with reference to the average value of the mixed venous blood oxygen saturation measured by a Swan-Ganz catheter and the average value of the oxygen saturation at the central vein ^[9]^. Veins seemed to have larger S-factor variability depending on position than arteries but had a consistent S-factor when analysed separately for each continuous venous system (Fig. 3).

***Supplementary Movie***

***Supplementary Video 1***

A video visualising the PA image of a palm, including the S-factor image.

***Supplementary Video 2***

A video visualising the PA image of perforators in the anterolateral thigh, including the S-factor image.

***Supplementary Video 3***

A video visualising the S-factor image obtained from two PA images measured at 756 and 797 nm. The extracted US lesion is overlaid as a red volume.

***Supplementary Video 4***

A video visualising the PA image reconstruction in a wide area. An image was created for each laser shot, and the entire image was constructed by adding these images.

***Supplementary Figures***

(a)

(b)　　　　　　　　　　　　　　　　　　 (c)

　

***Supplementary Figure 1***

(a)

(d)


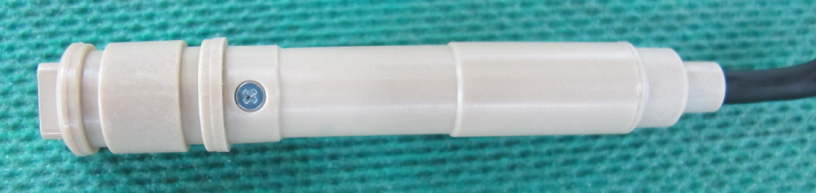


***Supplementary Figure 2***

a

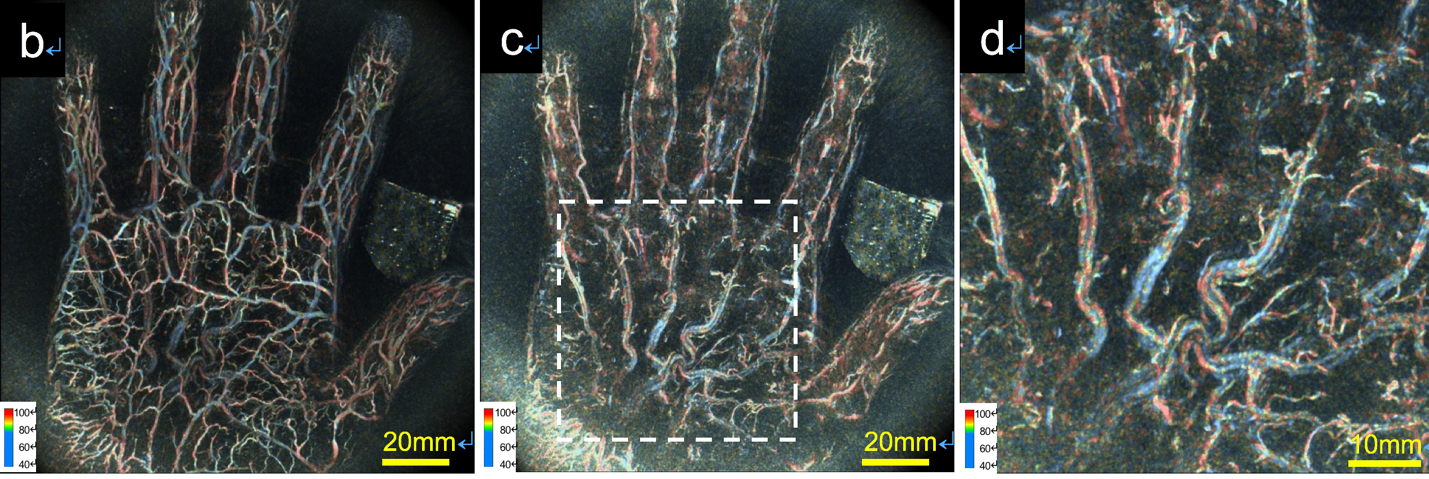


***Supplementary Figure 3***

***Supplementary Figure 4***

***Supplementary Figure 5***

***Supplementary Figure 6***

***Supplementary Figure 7***

***Supplementary Table 1***

(%)
